# Evaluating psychological interventions in a novel experimental human model of anxiety

**DOI:** 10.1016/j.jpsychires.2015.02.001

**Published:** 2015-04

**Authors:** Ben Ainsworth, Jemma E. Marshall, Daniel Meron, David S. Baldwin, Paul Chadwick, Marcus R. Munafò, Matthew Garner

**Affiliations:** a School of Psychology, University of Southampton, UK; b Clinical and Experimental Sciences, Faculty of Medicine, University of Southampton, UK; c Department of Psychology, King's College London, UK; d MRC Integrative Epidemiology Unit (IEU), The University of Bristol, UK; e UK Centre for Tobacco and Alcohol Studies and School of Experimental Psychology, University of Bristol, UK

**Keywords:** Mindfulness, Anxiety, Experimental medicine, Attention

## Abstract

Inhalation of 7.5% carbon dioxide increases anxiety and autonomic arousal and provides a novel experimental model of anxiety with which to evaluate pharmacological and psychological treatments for anxiety. To date several psychotropic drugs including benzodiazepines, SSRIs and SNRIs have been evaluated using the 7.5% CO_2_ model; however, it has yet to be used to evaluate psychological interventions. We compared the effects of two core psychological components of mindfulness-meditation (open monitoring and focused attention) against general relaxation, on subjective, autonomic and neuropsychological outcomes in the 7.5% CO_2_ experimental model.

32 healthy screened adults were randomized to complete 10 min of guided open monitoring, focused attention or relaxation, immediately before inhaling 7.5% CO_2_ for 20 min. During CO_2_-challenge participants completed an eye-tracking measure of attention control and selective attention. Measures of subjective anxiety, blood pressure and heart rate were taken at baseline and immediately following intervention and CO_2_-challenge.

OM and FA practice reduced subjective feelings of anxiety during 20-min inhalation of 7.5% CO_2_ compared to relaxation control. OM practice produced a strong anxiolytic effect, whereas the effect of FA was more modest. Anxiolytic OM and FA effects occurred in the absence of group differences in autonomic arousal and eye-movement measures of attention.

Our findings are consistent with neuropsychological models of mindfulness-meditation that propose OM and FA activate prefrontal mechanisms that support emotion regulation during periods of anxiety and physiological hyper-arousal. Our findings complement those from pharmacological treatment studies, further supporting the use of CO_2_ challenge to evaluate future therapeutic interventions for anxiety.

## Highlights

• 32 adults randomized to mindfulness/relaxation interventions before 7.5% CO_2_-challenge.
• Mindfulness practice reduced subjective anxiety compared to relaxation control.
• Open-monitoring achieved the greatest reduction in experimentally induced anxiety.
• There were no group differences in autonomic arousal or attention control.

## 1 Introduction

Inhalation of air enriched with 7.5% carbon dioxide (CO_2_) for 20 min increases self-report anxiety (e.g. worry, nervousness and tension) and autonomic arousal (e.g. heart rate and blood pressure) and provides a safe and reliable experimental model of anxiety in healthy humans (Bailey et al., 2005). The subjective and autonomic effects of 7.5% challenge are well characterised and are quantitatively and qualitatively less pronounced than the panic symptoms elicited by the 35% CO_2_ model of panic (see Colasanti et al., 2008). Recent studies suggest that in healthy individuals 7.5% CO_2_ inhalation can also induce a range of neuropsychological biases in attention and emotion processing that characterize clinical anxiety (review by Ainsworth and Garner, 2013). For example, 7.5% CO_2_ challenge impairs attention control and increases distractibility to environmental threat cues in eye-tracking tasks (Garner et al., 2011), and increases hyper-vigilance (Garner et al., 2012). Consequently 7.5% CO_2_ challenge is considered a putative human experimental model of subjective, autonomic and neuropsychological features of generalized anxiety, that can help evaluate therapeutic interventions, prior to phase-II/III clinical trials in patient populations (Bailey et al., 2011a).

Initial validation studies have examined whether established, licenced pharmacological treatments for anxiety can reduce CO_2_-induced anxiety in healthy humans. Single doses of the benzodiazepines lorazepam (2 mg) and alprazolam (1 mg) reduce CO_2_-induced subjective worry and anxiety (Bailey et al., 2007, 2009), as does a 3-week course of the selective serotonin reuptake inhibitor (SSRI) paroxetine (Bailey et al., 2007). A 7-day course of the corticotropin-releasing factor antagonist R317573 also reduces subjective response to CO_2_ (Bailey et al., 2011b), whilst pregabalin and the serotonin-noradrenaline reuptake inhibitor venlafaxine have more modest effects (Diaper et al., 2013, see Bailey et al., 2011a, 2011b Table 3 for review). These findings suggest future studies might use the CO_2_ model to examine the potential therapeutic efficacy of other novel treatments for anxiety, including psychological interventions.

Current treatment guidelines recommend cognitive-behavioural therapies (CBT) as the first-line psychological treatment for mild to moderate anxiety. While meta-analyses highlight the clinical effectiveness of CBT (Butler et al., 2006), access to CBT is limited by long waiting lists and variation in local services (in part due to cost of service delivery). Consequently, there is growing interest in the use of affordable, low-intensity psychological interventions for mild-moderate generalized anxiety that can more easily be delivered in group-settings, and practiced at home with remote/on-line support (Khoury et al., 2013). Of these, mindfulness/meditation-based interventions offer initial promise for a range of physical and neuropsychiatric conditions, including stress and anxiety (see Chen et al., 2012 for a meta-analysis of randomised controlled trials).

Mindfulness meditation techniques encourage deliberate, objective/non-judgemental attention to internal and external stimuli in the present moment (Williams and Kabat-Zinn, 2011). Such techniques are often incorporated into 8-week Mindfulness-based Stress Reduction (MBSR: Kabat-Zinn, 2003) interventions (to reduce stress) and Mindfulness-based Cognitive Therapy (MBCT: Teasdale et al., 2000), a version of MBSR developed for patients with recurrent depression. MBSR can reduce physiological responses to stress, including blood pressure (Carlson et al., 2007; Palta et al., 2012) and salivary cortisol (Carlson et al., 2007; Jensen et al., 2012). Briefer mindfulness interventions (3 × 1-h sessions) can reduce heart-rate in healthy volunteers (Zeidan et al., 2010). Mindfulness-meditation can also improve cognition in healthy populations (see Chiesa et al., 2011, for a review), including some cognitive processes that are perturbed in clinical anxiety (e.g. deficits in attention control, Ainsworth et al., 2013). However, to optimise mindfulness-meditation interventions for anxiety we need to isolate active component processes that mediate *anxiolytic* response. Here we report findings from the first study to use a healthy human experimental model of anxiety to evaluate and compare two core psychological components of mindfulness meditation: focussed attention and open monitoring.

Neuropsychological models suggest focused attention and open-monitoring are distinct components of mindful meditation (Lutz et al., 2008; Manna et al., 2010; Holzel et al., 2011). Focused attention (FA) involves restricting awareness to a volitionally chosen object (e.g. localised sensation of breathing) and engaging in ‘self-monitoring’ for unwanted intrusive thoughts and distractions. In contrast, open monitoring (OM) encourages active monitoring and acceptance of internal and external sensation to promote a receptive field of non-judgemental awareness. By encouraging awareness of internal emotional experiences, yet recognising them as subjective and prone to personal bias, OM emphasizes affective/attitudinal facets, in contrast to FA, in which attentional skills are more prominent.

In the present study healthy volunteers recruited from the community were randomized to one of three intervention conditions (FA, OM, relaxation control) immediately before completing 7.5% CO_2_-challenge and associated self-report measures of anxiety, autonomic arousal and attention control (eye-tracking antisaccade task, Garner et al., 2011). We predicted that OM and FA (compared to relaxation control) would reduce self-report anxiety, autonomic arousal and attention to threat during CO_2_-challenge. The existing literature further suggests that OM and FA may target different features of CO_2_-induced anxiety, with OM having a greater effect on the subjective affective experience of CO_2,_ whereas FA might achieve greater effects on attention control during CO_2_ challenge.

## 2 Method

### 2.1 Participants

10 male and 22 female healthy young adults with no prior formal experience of mindfulness meditation were recruited through adverts placed around the university campus and local community (mean age = 21.7 years, SD = 3.2). A structured diagnostic interview based on DSM-IV criteria (Mini International Neuropsychiatric Interview – MINI; Sheehan et al., 1998) was used to screen eligible participants. Exclusion criteria included prior experience with mindfulness meditation, recent use of medication (during the past eight weeks, except for topical treatments; occasional aspirin or paracetamol; oral, injectable or skin patch contraception), pregnancy, history of asthma/respiratory illness, high blood pressure (>140 systolic and/or 90 diastolic), cardiovascular disease, migraines, current or lifetime history of psychiatric illness (including lifetime history/family history of panic attacks), regular smoker (more than 6 cigarettes/day), under- or over-weight (body mass index less than 18 or greater than 28 kg/m^2^), current or past drug or alcohol dependence and recent use of illicit drugs (during previous 8 weeks) or alcohol (verified by breath test). Eligible participants were randomly assigned to one of three experimental groups in a single-blind between-group design: focused attention meditation (FA: *N* = 11), open monitoring meditation (OM: *N* = 11), and a relaxation control (RC: *N* = 10). Participants received course credits or financial compensation for time spent (£20).

### 2.2 Methods and procedure

The study protocol was approved by the Ethics and Research Governance office at the University of Southampton and was in accordance with the 2008 Declaration of Helsinki. All participants provided informed consent prior to participation.

Participants attended a single test session and first completed established standardized self-report measures of trait anxiety (STAI-trait: Spielberger et al., 1983), attention control (Attention Control Scale, ACS: Derryberry and Reed, 2002) and dispositional trait mindfulness (Mindful Attention Awareness Scale, MAAS: Brown and Ryan, 2003).

The following primary outcome measures were taken at baseline, immediately post-intervention and immediately after 7.5% CO_2_ challenge: heart rate (BPM), diastolic and systolic blood pressure (Omron-M6 arm-cuff monitor, Medisave, UK), and visual analogue ratings quantifying the extent to which participants felt ‘anxious’, ‘nervous’, and ‘worried’ (response scale ranged from ‘Not at all’ (0) to ‘Extremely’ (100)). To allow comparison with previous studies we administered the state version of the STAI (Spielberger et al., 1983), and the Positive and Negative Affect Scale (PANAS; Watson et al., 1988) at baseline, and immediately following 7.5% CO_2_ challenge. These lengthier measures were not administered post-intervention in order to limit the delay between completing the intervention and commencing the CO_2_ challenge. All self-report measures demonstrated good internal consistency (α's > 0.74).

### 2.3 Psychological interventions

Each intervention lasted 10 min and was developed and recorded by a consultant psychiatrist (DM) with clinical expertise in delivering mindfulness-based interventions (since 2000) and several thousand hours of personal mindfulness practice (since 1990). We have used these interventions in previous studies to reveal effects of FA and OM on executive attention in healthy individuals (Ainsworth et al., 2013).

In the focused attention meditation, participants were asked to “Find a place where the sensations of your breath are particularly clear right now…at the tip of the nose, the back of the throat, the chest or the abdomen”….“Make a decision to stay with this place for the duration of this exercise rather than moving your awareness from one place to another.”….“Turn your awareness towards this place…allowing your awareness to settle on this point…allowing the mind to become comfortable here”….“Maintain this focus, and if the mind wanders, gently return the mind to this place.”…. “If you find your mind has wandered, lightly and firmly return your focus to this place….”“Examining the sensation of the breath, and making the focus of attention as fine and as exact as possible – really pinpoint this one point where the breath is observed.”

In contrast, in the open monitoring meditation participants were asked to “Allow a sense of awareness of the breath and physical sensations in the body generally to gradually expand”….“Allowing your focus to include the sounds that you're hearing, whatever the eyes see, and perhaps any smells, to come within your field of awareness.”…“Sitting here, with all of this, perhaps allowing your emotional tone, how you are feeling right now, to become part of this field of awareness – whatever sense of comfort or discomfort, any emotions you feel right now, allowing that to become part of your field of awareness right now, noticing any changes that may occur”. Control participants were asked to sit quietly and relax. Participants subsequently completed post-intervention measures followed by the CO_2_ challenge.

*7.5% CO*_*2*_ *challenge*: Participants inhaled air enriched with 7.5% CO_2_ (21% O_2_, balance N_2_) for 20 min through an oral-nasal face mask. Midway through the inhalation participants completed an emotional version of the antisaccade eye-movement task in which they were instructed to look towards (prosaccade) or look away from (antisaccade) 8 negative and 8 neutral pictures selected from the standardized International Affective Picture Set (see Garner et al., 2011 for details). This task provides measures of attention control (i.e. ability to inhibit eye-movements to pictures on antisaccade trials) and selective attention (i.e. speed and likelihood of looking towards negative relative to neutral stimuli). Participants completed 96 trials that were presented in a random order (24 trials per saccade-type × picture valence condition). Trials were counter-balanced for stimulus location. The task was presented using Inquisit 2 computer software. Consistent with Garner et al. (2011) horizontal eye-movements were measured by electro-oculography and sampled at 1000 Hz (MP150-amplifier and AcqKnowledge 3.8.1 software, Biopac systems, Goleta, CA).

## 3 Results

### 3.1 Group characteristics

One-way ANOVAs confirmed that groups did not differ in self-report trait anxiety, attention control or mindfulness (see Table 1). Scores were within the range typical of healthy non-clinical samples (e.g. Garner et al., 2011). A Freeman-Halton extension of Fisher's exact test confirmed that groups did not differ in gender, *p* = .99, nor age, *F*_(2,29)_ = .90, *p* = .42.

### 3.2 Effects of FA and OM on subjective anxiety

**Fig. 1:**
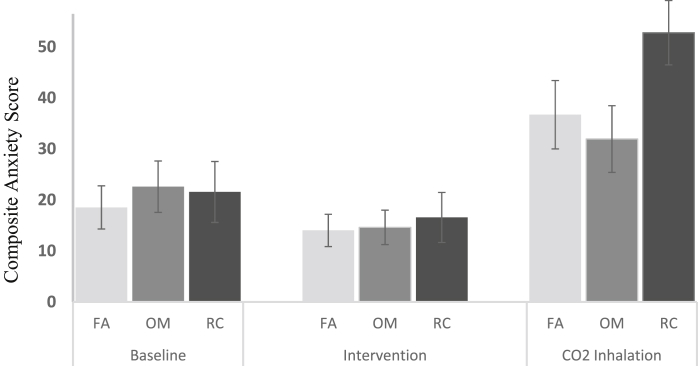
Effects of FA, OM and RC on CO_2_ increased anxiety, *F*_(4,58)_ = 3.19, *p* = .020 $\eta_{P}^{2}$ = .18, *90% CI* = .02 to .28.

Visual analogue ratings were averaged to provide a composite anxiety score and entered into a mixed design ANOVA with Group (FA, OM, RC) as a between-subjects factor, and Time (baseline, post-intervention, and post-CO_2_) as a within-subjects factor. Results revealed a Group × Time interaction [*F*_(4,58)_ = 3.19, *p* = .020, $\eta_{P}^{2}$ = .18, *90% CI* = .02 to .28], see Fig 1. Follow-up analyses tested whether intervention groups differed in anxiety i) immediately after the intervention, and ii) following CO_2_ challenge, relative to baseline. Participants in each group reported lower levels of anxiety following their intervention compared to baseline (*p*'s ≤ .05), but the magnitude of this reduction did not differ across groups [*F*_(2,29)_ = 0.69, *p* = .51]. Consequently all three interventions produced comparable acute improvements in subjective mood. However in contrast, and as predicted, intervention groups differed in their response to CO_2_-challenge [*F*_(2,29)_ = 4.42, *p* = .021, $\eta_{P}^{2}$ = .23, *95% CIs* = .003 to .430]. The OM group experienced the smallest increase in anxiety following CO_2_-challenge [*M* = 9.35, *t*_(10)_ = 2.08, p = .06, 95% CI = 19.38 to −0.68, *d*_*z*_ = 0.62], followed by the FA group [*M* = 18.16, *t*_(10)_ = 4.01, 95% CI = 28.26 to 8.07, *p* = .002, *d*_*z*_ = 1.21], while the relaxation control group experienced the largest increase in anxiety [*M* = 31.20, *t*_(9)_ = 4.81, *p* = .001, 95% CI = 45.87 to 16.53, *d*_*z*_ = 1.52]. Between-group comparisons provide evidence that OM participants experienced less anxiety post-inhalation than the relaxation control group [*t*_(19)_ = 2.28, *p* = .034, *d*_*s*_ = 0.99, 95% CI = 1.70 to 39.90], with some evidence that FA had a more modest effect on CO_2_-induced anxiety compared to the relaxation control group [*t*_(19)_ = 1.74, *p* = .098, *d*_*s*_ = 0.76, 95% CI = −2.9 to 35.0], see Fig. 1.

Analyses of secondary measures taken at baseline and post-inhalation revealed large Group × Time effects on STAI-state anxiety [*F*_(2,29)_ = 5.13, *p* = .012, $\eta_{P}^{2}$ = .26, 90% CI = .04 to .42] but weaker effects on positive and negative affect, see Table 1 [*F*_(2,29)_ = 3.67, *p* = .038, 90% CI = .01 to .37, $\eta_{P}^{2}$ = .26; *F*_(2,29)_ = 2.75, *p* = .081].

### 3.3 Effects of FA and OM on heart rate and blood pressure

Measures of heart rate and mean arterial pressure (2 × DBP + SBP)/3) were entered into separate mixed design ANOVA with Group (FA, OM, RC) as a between-subjects factor, and Time (baseline, post-intervention, and post-CO_2_) as a within-subjects factor. CO_2_ challenge increased heart rate [*F*_(2,29)_ = 24.14, *p* < .001, $\eta_{P}^{2}$ = 0.62, *90% CI* = .40 to .71] but this increase did not differ across groups [*F*_(4,58)_ = 1.17, *p* = .33]. CO_2_ challenge also produced robust increases in blood pressure in each group [*F*_(2,29)_ = 16.55, *p* < .001, $\eta_{P}^{2}$ = .53, *90% CI* = .28 to .64]. A Group × Time interaction [*F*_(4,58)_ = 3.05, *p* = .024, $\eta_{P}^{2}$ = .17, *90% CI* = .01 to .27] suggests that this pattern differs across group, however post-hoc tests suggest that this was driven by comparatively high BP in the RC group at baseline, rather than group differences in the extent to which CO_2_ increased blood pressure (mean FA vs RC difference at baseline of 6.43 [*t*_(19)_ = 1.97, *p* = .06]; no other between-group differences at any time [ts < 1.26, *ps* > .22]).

### 3.4 Effects of FA and OM on antisaccade performance during CO_2_ challenge

The direction and latency of eye-movements were scored manually using AcqKnowledge software and blind to trial-type and group membership, consistent with Garner et al., 2011. Anti-saccade error rates and latencies were entered into separate mixed-design ANOVA with group, and stimulus valence (negative vs. neutral) as independent variables. There was evidence that participants made more erroneous eye-movements towards neutral relative to negative pictures [*F*_(1,28)_ = 10.21, *p* < .03, $\eta_{P}^{2}$ = .27], however the omnibus analysis did not reveal clear effects of group (nor interactions with group), *F*s < 1.72, *p*s > .197.

## 4 Discussion

Our findings are the first to show that psychological techniques employed in contemporary mindfulness-meditation interventions can alleviate anxiety in an experimental healthy human experimental model of anxiety. FA and OM practice reduced subjective feelings of anxiety during 20-min inhalation of 7.5% CO_2_ compared to relaxation control. OM practice produced a strong anxiolytic effect, whereas the effect of FA was more modest. Anxiolytic effects of OM and FA occurred in the absence of group differences in autonomic arousal – all three groups experienced large and comparable increases in heart rate and mean arterial blood pressure following CO_2_-challenge. Contrary to predictions, neither OM nor FA affected eye-tracking measures of attention control and selective attention during CO_2_-challenge. Consequently OM, and to a lesser extent FA, appear to have a selective effect on CO_2_-induced subjective feelings of anxiety, but not autonomic or neuropsychological consequences of CO_2_-challenge.

Consistent with mechanisms emphasized by recent neuropsychological models of mindfulness meditation, our OM intervention guided participants to regulate emotion through positive reappraisal, exposure, extinction and reconsolidation; that is, to embrace whatever is present in the field of awareness but approach ongoing emotional reactions non-judgmentally and with acceptance of bodily and affective responses (see Holzel et al., 2011). In contrast, FA practice encouraged non-appraisal (rather than re-appraisal) through strict regulation of attention, and may have achieved only modest anxiolytic effects through avoidance/suppression of affective and physiological reactions. Recent evidence suggests that individuals who report a dispositional tendency to ‘restrict attention to the present moment’ are likely to experience less anxiety when actively suppressing the effects of a 90 s 15% CO_2_ inhalation challenge (Bullis et al., 2014). Together, these findings suggest FA may promote suppression to reduce anxiety during stress, whereas OM might activate a range of regulatory mechanisms to achieve greater anxiolysis.

Might the anxiolytic effects of OM and FA reflect greater immediate improvements in mood that sustain throughout the CO_2_-challenge (i.e. residual carry-over effects)? Our results suggest not – all three interventions produced comparable improvements in mood from baseline to post-intervention, and there was no evidence that baseline or post-intervention levels of anxiety moderated the effects of OM and FA on CO_2_-induced anxiety. A more parsimonious explanation is that OM, and to a lesser extent FA, enabled participants to better regulate emotional experience during CO_2_-challenge and autonomic hyper-arousal.

The effects of OM and FA in this model complement those of established pharmacological treatments for anxiety. Benzodiazepines and selective serotonin reuptake inhibitors both reduce 7.5% CO_2_-induced subjective anxiety, but not autonomic arousal (Bailey et al., 2007, 2009). Conversely drugs that target somatic mechanisms, such as the non-selective adrenergic beta-blocker propranolol do not reduce CO_2_-induced anxiety despite lowering CO_2_-increased heart rate (Papadopoulos et al., 2010). Together these findings suggest that psychological and pharmacological interventions may un-couple established associations between subjective and autonomic responses to CO_2_-challenge (Garner et al., 2011, 2012; Pinkney et al., 2014), and may do so through similar central neural mechanisms. For example, the subjective effects of several classes of anxiolytic drugs are in-part mediated through prefrontal down-regulation of sub-cortical mechanisms implicated in anxious responding (e.g. amygdala and locus-coeruleus). Likewise, mindfulness training is associated with increased prefrontal activity and prefrontal-amygdala connectivity, and corresponding alleviation of symptoms in patients with generalized anxiety disorder (Holzel et al. 2011). Comparable effects of anti-panic medication and cognitive behaviour therapy on subjective response to CO_2_-challenge in patients with panic disorder support a common anxiolytic pathway (Gorman et al., 2004).

In the current study we extended the traditional 7.5% CO_2_ model of anxiety to include an eye-tracking measure of attention control and selective attention. Contrary to predictions, neither FA nor OM improved antisaccade performance; rather, all three groups were characterized by poor performance, with observed antisaccade error rates comparable with those observed in previous CO_2_-challenge studies (Garner et al., 2011). Previous studies suggest that acute interventions can increase attention control in unchallenged healthy individuals (Dickenson et al., 2013), but that prolonged practice can achieve larger improvements in cognitive control (Ainsworth et al., 2013) and autonomic arousal (Jensen et al., 2012) in unchallenged healthy populations, albeit in the absence of changes in mood. Cross-sectional studies also suggest that individuals who report elevated trait/dispositional mindfulness exhibit reduced anxiety during social stress paradigms, (Brown et al., 2012), and that only individuals with high levels of trait mindfulness exhibit a comprehensive subjective and physiological anxiolytic response to short mindfulness interventions (e.g. 3 sessions × 25 min; Creswell et al., 2007). Our study was not powered to examine the effect of trait mindfulness nor other trait characteristics on response to FA/OM, and it is not surprising that supplementary analyses did not identify trait predictors of response to intervention, nor CO_2_-challenge. Accordingly, future studies should identify predictors of subjective, autonomic and neuropsychological response to brief and longer-term psychological interventions in general, and particularly during periods of anxiety (as modelled in the present study). To this end, the 7.5% CO_2_ model of anxiety is well placed to efficiently evaluate and help optimize novel anxiolytic interventions for anxiety in healthy, vulnerable/at-risk and clinical populations.

## Funding

Medical Research Council [MR/J011754/1] awarded to MG, MRM and DSB.

BA was funded by an interdisciplinary Medical Research Council/Economic Social Research Council studentship [ES/H018514] awarded to MG, BA, PC and DSB.

The funding source(s) had no role on the design, collection, analysis or interpretation of the data, the writing of the report nor the decision to submit the article for publication.

## Contributors

BA was involved in study design, data collection, data analysis, interpretation and writing of the report.

JM was involved in data collection and data analysis.

DM was involved in study design, data collection and writing of the report.

PC was involved in study design and writing of the report.

DSB was involved in study design, data collection and writing of the report.

MM was involved with study design and writing of the report.

MG was involved in study design, data analysis, interpretation and writing of the report.

All authors have approved the final submitted article.

## Conflicts of interest statement

None declared.

## Figures and Tables

**Table 1:** Group characteristics.

Trait characteristics				
	Focused Attention (FA)	Open-monitoring (OM)	Relaxation control (RC)	One-way ANOVA
STAI	33.5 (6.5)	35.0 (5.5)	33.4 (5.0)	*F*_(2,29)_ = .28, *p* = .7*6*
MAAS	61.8 (6.6)	55.8 (6.9)	57.9 (6.5)	*F*_(2,29)_ = 2.26, *p* = .12
ACS	49.2 (8.2)	52.4 (5.7)	50.7 (8.5)	*F*_(2,28)_ = .46, *p* = .63

**Mean autonomic scores across time (baseline vs. post-intervention vs. post-inhalation)**										
	FA			OM			RC			
	Base	Post-Int.	Post-CO_2_	Base	Post-Int.	Post-CO_2_	Base	Post-Int.	Post-CO_2_	Group × Time ANOVA
MAP	83.2 (6.7)	86.7 (12.2)	94.9 (9.5)	84.8 (9.4)	83.8 (9.1)	91.3 (12.5)	89.7 (8.2)	81.7 (10.3)	90.6 (10.8)	*F*_(4,58)_ = 3.05, *p* = .024, $\eta_{P}^{2}$ = .17
HR	68.0 (9.5)	70.4 (10.3)	90.0 (18.0)	73.1 (16.1)	70.8 (17.9)	83.0 (28.4)	74.2 (11.7)	71.5 (11.4)	86.1 (10.8)	*F*_(4,58)_ = 1.17, *p* = .33

**Mean antisaccade error-rates and latencies by valence (negative vs. neutral)**							
	FA		OM		RC		
	Neg.	Neut.	Neg.	Neut.	Neg.	Neut.	Group × emotion ANOVA
Error-rate	.56 (.22)	.61 (.22)	.56 (.23)	.59 (.22)	.42 (.17)	.56 (.22)	*F*_(4,58)_ = 1.17, *p* = .33
Latency	183.1 (45.6)	182.4 (57.8)	168.4 (23.0)	190.8 (39.5)	178.7 (20.4)	183.4 (40.2)	*F*_(4,58)_ = 1.29, *p* = .29

Note: STAI = Spielberger Trait Anxiety Inventory, MAAS = Mindfulness Attention Awareness Scale, ACS = Attention Control Scale, MAP = Mean Arterial Pressure, HR = Heart-Rate.
